# Grandparent–Grandchild Coresidence Among Middle-Aged and Older Adults Around the Globe

**DOI:** 10.3390/populations1020012

**Published:** 2025-06-06

**Authors:** Sarah Anne Reynolds, Ryan Edwards, Jacqueline M. Torres

**Affiliations:** 1 Berkeley Population Center, University of California, Berkeley, CA 94720, USA; 2 Department of Epidemiology and Biostatistics, University of California, San Francisco, CA 94158, USA

**Keywords:** intergenerational coresidence, older adults, living arrangements, household structure

## Abstract

Although the relationship between grandparent and grandchild is often unique due to the supportive and foundational roles grandparents can have in the lives of young or youthful grandchildren, the extent of grandparent–grandchild coresidence globally is under-researched. We harmonized household roster survey data on grandchild coresidence using population-based data on adults 55+ years across 24 countries. Grandchild coresidence rates ranged from 41.1% in Mexico to 0.1% in Sweden. Across contexts, grandchild coresidence was more common among women (compared to men), non-partnered individuals (compared to partnered individuals), those who reported difficulty with activities of daily living (compared to those without such difficulties), and those with education levels below the median (compared to those above the median). Logit regressions indicated gaps in rates of grandchild coresidence by partner status, ADL status, and education were generally not driven by income or other socio-demographic variables. Coresidence with adult grandchildren was not uncommon in most countries with non-negligible rates of grandchild coresidence. In about 25% of households of middle-aged and older adults coresiding with grandchildren, grandchildren ages 0–5 years were present. Future research should consider the meaning of grandparent–grandchild coresidence for the health outcomes of middle-aged and older adults globally, particularly when grandparents are not caregivers of grandchildren.

## 1. Introduction

Research on intergenerational coresidence dynamics among older adults focuses mainly on coresidence with adult children. Grandchildren, however, can play a unique role in the lives of middle-aged and older adults. Grandparent–grandchild coresidence is becoming more common in the US [1] and multi-generational households are prevalent around the world [2]. Across many cultural contexts, the relationship between grandparent and grandchild is often distinct from that between parent and adult child due to the supportive and foundational roles grandparents can have in the lives of young or youthful grandchildren. Grandparents often take on a supportive role without having to be a disciplinarian [3], resulting in relationships with grandchildren that may be less conflictive and more affectionate than those that middle-aged and older adults had with their own children. Thus, grandchildren may perceive their grandparents as confidants and companions [4]. In the context of high rates of loneliness that are associated with health declines [5,6], the meaning provided from these grandparent–grandchild relationships could be crucial for middle-aged and older adult’s well-being [7–9].

The grandparent–grandchild relationship may also differ from the parent–child relationship in other ways. Coresident adult children generally may have capacity to contribute financially or through domestic labor to the household and generally do not require extra caretaking. Grandchildren—who are often too young to participate in the labor force and may need more care at home as well—could require time or financial support from grandparents who coreside in the household [10].

Despite the theoretical importance for the well-being of middle-aged and older adults, the extent of grandparent–grandchild coresidence globally is under-researched. We fill this gap by estimating the prevalence of grandchild coresidence among middle-aged and older adults across 24 countries using harmonized population-based data. We additionally examine how rates differ across dimensions of socio-economic disadvantage and health status, and we characterize aspects of household structure and changes with age. The cross-national comparative nature of this study allows us to draw conclusions about the general nature of grandchild coresidence among middle-aged and older adults and to highlight some unique contexts which may require tailored family policy.

## 2. Literature Review

Much research has already been generated on the coresidence of older adults and their adult children [11–15]. By extension, if coresiding adult children have their own children, the motives for two-generation coresidence often apply to three-generation coresidence.

For example, intergenerational households are often described as the outcome of financial difficulties. A line of study is dedicated to examining “doubling up” in times of hardship. Household size is cyclical, with more families joining together under the same roof during recessions [16]. For example, the Great Recession accelerated intergenerational coresidence in multiple global contexts [11,17].

One of the financial benefits of intergenerational coresidence can be related to childcare. Grandparent coresidence for the purpose of grandchild care can often replace the lack of affordable childcare centers or parental work leave [18]. This arrangement can benefit the family if the younger generation has more earning potential than older adults who can take on caregiving. The younger generation may even migrate, leaving the grandchild in the sole care of the grandparent. These “skipped-generation” households are a special subset of families where grandparents have custody (though perhaps informally) of their grandchildren, potentially for financial reasons. There may be other contributors: drug abuse in the middle generation is a common contributor to the skipped generation structure in the United States [1].

The need for care and financial support can also go in the other direction. In the context of rising life expectancy and mixed evidence of morbidity compression in many global contexts, those in the grandparent generation may require support with instrumental and basic activities of daily living [19]. The limited availability of formal long-term services and supports in most global settings means that family members—and particularly those who coreside—provide the majority of late-life care [18]. Grandparents may receive important assistance from older (e.g., adolescent or adult) coresident grandchildren that may supplement or substitute care received from other coresident family members. In addition, prior research has shown that grandparents who coreside with grandchildren are more likely to be living in poverty than their counterparts without coresident grandchildren [20].

Grandchild coresidence may also result from family disruption, including marital dissolution. For example, widowhood may exacerbate financial and care-related needs for middle-aged and older adults and serve as a catalyst for intergenerational coresidence. Similarly, if an adult child experiences a separation, the adult child may join households with their parent(s), often to facilitate financial and caregiving support.

Factors that contribute toward intergenerational coresidence tend to influence older women more than older men. Globally, women have longer life expectancies, tend to live more years with late-life disability, and are less likely to be married or partnered. The motherhood pay gap—caused by taking time away from work and thus not gaining as many skills or wage increases—results in smaller lifetime earnings than men and thus potentially also smaller levels of savings and assets. In some locations, women may devote their entire lives to domestic labor: they are dependent on their spouses for market earnings.

## 3. Methods

### 3.1. Data

We used publicly available data from 24 countries collected from five population-based data sources on middle-aged and older adults that included detailed household rosters. We generally relied on harmonized code from the Gateway to Global Aging platform. Studies covered China (China Health and Retirement Longitudinal Study), Costa Rica (Costa Rican Longevity and Healthy Aging Study), the United States (Health and Retirement Study), Mexico (Mexican Health and Aging Study), and 20 European Countries (Survey of Health, Aging, and Retirement in Europe). For each study, we focused on the most recently completed wave of data that occurred prior to the COVID-19 pandemic. In many contexts, the COVID-19 pandemic led to changes in the prevalence of intergenerational coresidence that might not generalize to non-pandemic years. Citations and links to each study are found in the Data Availability Statement at the end of this article.

We limit the sample to directly sampled respondents (i.e., excluding spouses or partners) and limited participants to those 55 years+ in the survey year (some studies include respondents as young as 40 years old, although most start at 50 years+).

### 3.2. Grandchild Coresidence

Grandchild coresidence information had not previously been harmonized on the Gateway to Global Aging platform. We relied on data from detailed household rosters to generate binary variables for (1) any grandchild coresidence and (2) the age ranges of the youngest coresident grandchild (0–5, 6–17, and 18+). The youngest children generally require the most assistance in functional care activities (feeding, bathing, dressing, etc.), potentially directing middle generation time away from grandparent care. In addition to being more autonomous, children ages 6 and older do not require family supervision during the public-school day.

The data do allow for distinguishing between grandchildren and great-grandchildren, but we pool great-grandchildren into the grandchildren category. We expect similar relationships between grandparents and grandchildren as between grandparents and greatgrandchildren. Additionally, great-grandchildren appeared in households in only five countries (Austria, Italy, Mexico, Poland, and Slovenia). Mexico has the maximum rate, with 3% of 3+ generation households including great-grandchildren.

### 3.3. Socio-Demographic and Economic Measures

We used harmonized measures of respondent’s sex, partner status, and whether they had any difficulty with activities of daily living (ADLs). The variables urban/rural, education (above vs. below the median of the individual’s sex and age category within each country), and natural log of total household are proxies for socio-economic advantage. We calculated the presence and number of coresident children and coresident children-in-law from the household roster. Additional control variables used in some analyses were age and total number of children, coresident or not.

### 3.4. Analyses

For each country, we estimated the prevalence and 95% confidence intervals (CIs) of grandchild coresidence among adults aged 55+ using study-specific sampling weights. We repeated this exercise, stratifying by measures.

We then estimated the associations between socio-demographic measures and the odds of grandchild coresidence. We further examined three of the characteristics (partnered, ADL status, and high/low education) to examine if income and other covariates (sex, age, age squared, age cubed, urban/rural, and number of children) were driving the associations found between grandchild coresidence and these characteristics. We used logit regression (with population weights) and report margins in the regression tables in the Supplementary Materials for all countries, but for simplicity we only present graphs for six countries with the highest grandchild coresidence rates: Mexico, Costa Rica, China, Poland, Croatia, and the US.

To explore grandchild coresidence in the context of the larger household composition, we considered the subsample of middle-aged and older adults who had coresident grandchildren. For each country with more than 100 individuals in the sample with coresident grandchildren, we calculated the percentage—applying sampling weights—of these that have three-generation households, which means the middle generation (a child or child-in-law) is also present in the household. We contrasted this with the percentage of skipped-generation households where the grandchild coresides with a grandparent without the grandchild’s parent in the household.

Finally, we calculated the number of grandchildren and the ages of the grandchildren among the subsample of middle-aged and older adults who have coresident grandchildren. We examined the age profile of the youngest grandchild, groups those who were 0–5 years, 6–17 years, and 18+ years. In our graphs by age of the grandparent, we only considered ages 55–85 since there are few individuals aged > 85 years. With age as the running variable, we present polynomial smoothing functions (degree zero, Epanechnikov kernel, and bandwidth 5).

## 4. Results

**Figure 1.**
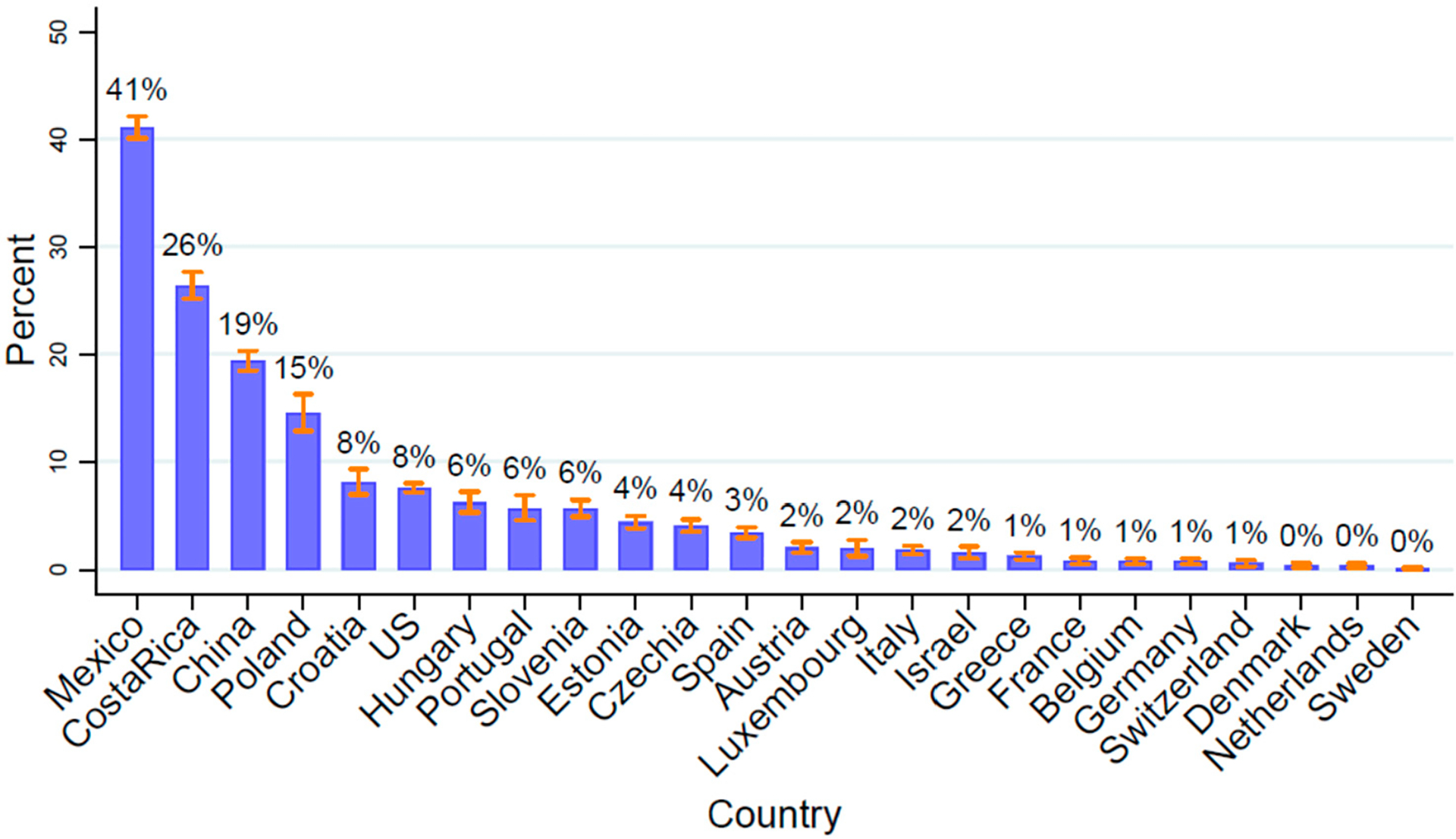
Older adults’ rates of coresidence with grandchildren vary widely across countries. Samples include older adults ages 55+ years. Data from the Gateway to Global Aging Studies; Grandchild coresidence calculated by the authors. Population weights applied. Data from 2015 with the following exceptions: China 2018, Costa Rica 2005 & 2011, Hungary 2011, Mexico 2012, Netherlands 2013, US 2014. See Supplementary Table S1 for numerics on 95% confidence intervals.

The prevalence of grandchild coresidence among adults aged 55+ ranged from 41.1% (CI 40.1–42.1%) in Mexico to 0.1% (CI 0.0–0.2%) in Sweden (Figure 1, Table S1). A quarter of middle-aged and older adults in Costa Rica and a fifth of middle-aged and older adults in China coresided with grandchildren. Rates in European countries were all lower than 10%, with the exception of Poland (14.5%, CI 12.8–16.3%). The US rate is 7.6% (CI 7.2–8.0%), indicating that over 5 million middle-aged and older adults in the US had coresident grandchildren as of 2014.

**Figure 2.**
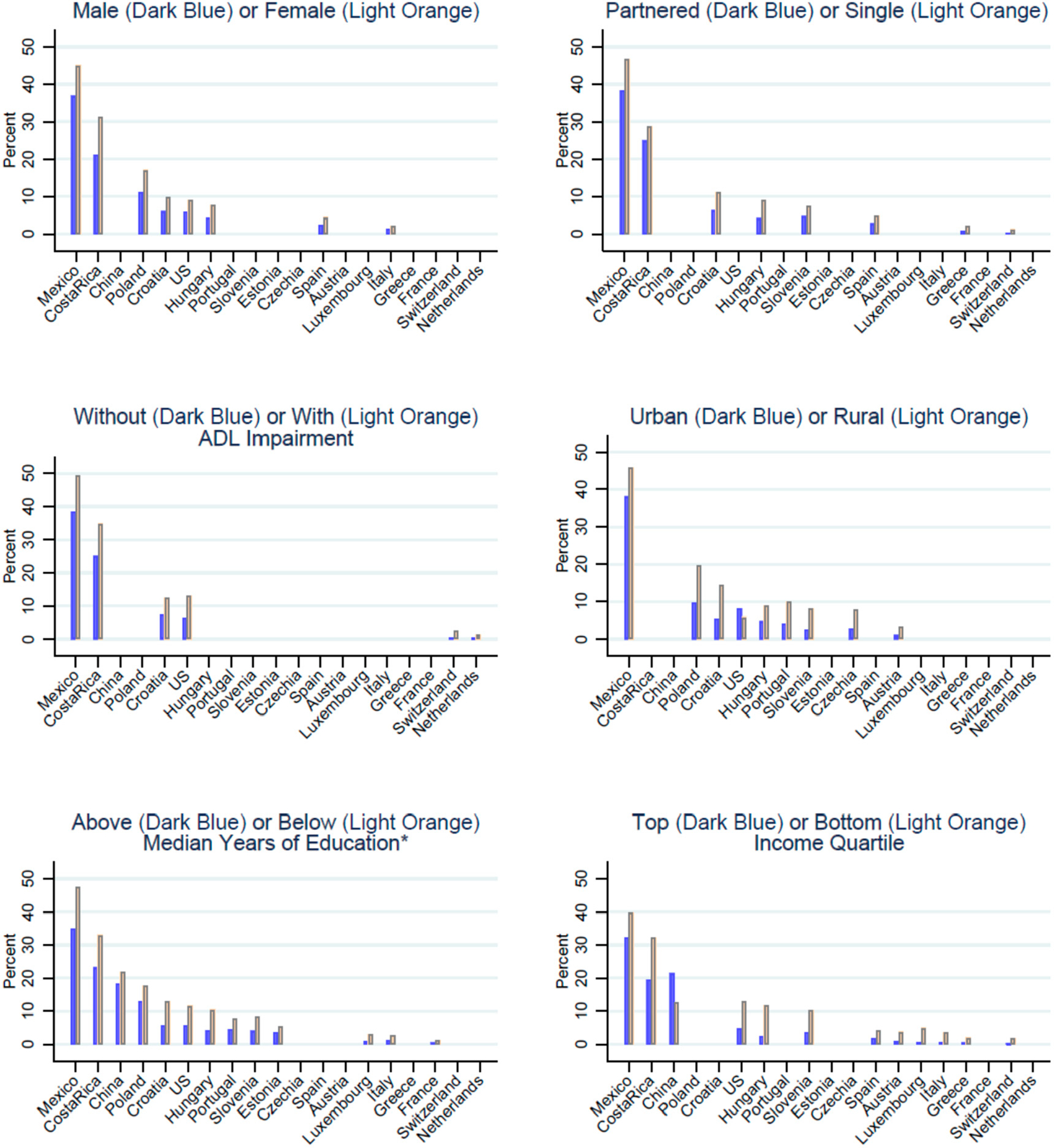
Differences in rates of coresidence with grandchildren by aspects of socio-demographic characteristics. Countries are omitted from the graphs if the difference is not statistically significant at 95%. Samples include older adults ages 55+ years. ADL—Activities of Daily Living. Data from the Gateway to Global Aging Studies; Grandchild coresidence calculated by the authors. Population weights applied. Data from 2015 with the following exceptions: China 2018, Costa Rica 2005 & 2011, Hungary 2011, Mexico 2012, Netherlands 2013, US 2014. See Supplementary Table S1 for exact rates and confidence intervals of all countries.

Only five countries did not have any statistically significant differences across all the heterogenous aspects we examined: Belgium, Denmark, Germany, Israel, and Sweden. However, due to low prevalence of grandchild coresidence in these countries (2% or less), that finding may be due to the small sample size of middle-aged and older adults coresiding with grandparents, resulting in very little statistical power to detect differences. No countries had statistically significant differences in all six aspects, but five countries had statistically significant differences in five of the aspects that we examined: Costa Rica, Croatia, Hungary, Mexico, and the US. Our graphs show rates contrasting each heterogeneity if the differences were statistically significant within the country (Figure 2). Table S1 includes confidence intervals for all countries and aspects. Being relatively less educated appears to be most commonly associated with coresident grandchildren in our sample. Differences by ADL status were less common: there was evidence of statistically significant heterogeneity in only five countries. For each aspect, most countries follow the same pattern, with the same group having a higher rate than the other whenever there is a statistically significant difference. For example, in all countries where there is a significant difference by the aspect of sex, females have a higher rate of grandchild coresidence than men. This holds for all aspects examined, with two exceptions. In the US, the urban rates of grandchild coresidence are higher than the rural rates (8.2% CI 7.7–8.7% and 5.9% CI 5.2–6.6%, respectively), and in China, the top income quartile have a higher rate of grandchild coresidence than the bottom quartile (21.4% CI 19.1–23.7% and 12.5% CI 10.7–14.4%, respectively).

**Figure 3.**
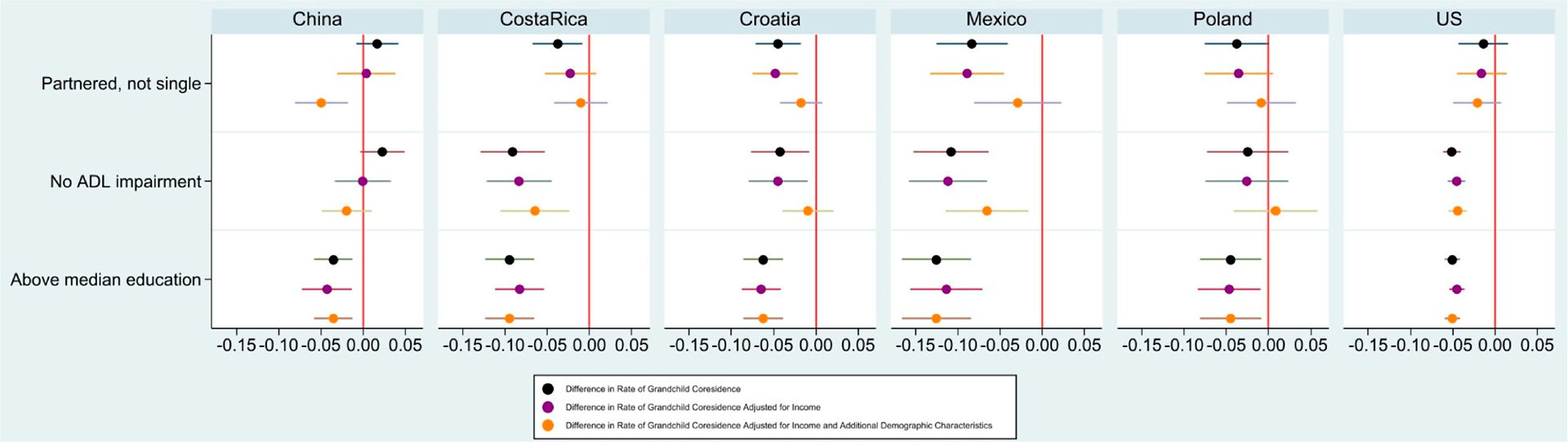
Income and demographic variables explain little of the gap in grandchild coresidence by partner status, ADL impairment, and high/low education. Adults ages 55–85 years. Data from the Gateway to Global Aging Studies; Grandchild coresidence calculated by the authors. Population weights applied. Negative estimates indicate the group has a lower rate of grandchild coresidence than their corresponding group. Partnered, not single corresponds to Partnered, single. No ADL impairment corresponds to ADL impairment. Above median education corresponds to below median education. Median education determined by the country, sex, and age group of the individual. Additional Demographic Characteristics are Sex, Age, Age Squared, Age Cubed, Number of Children, and Urban/Rural.

Our further examination of the gaps in rates of grandchild coresidence by partner status, ADL status, and education revealed that these gaps were generally not driven by income or other socio-demographic variables (sex, age, age squared, age cubed, urban/rural, and number of children): of the thirteen prevalence gaps that were statistically significant prior to any adjustment in the six countries with the highest rates of grandchild coresidence among middle-aged and older adults (China, Costa Rica, Croatia, Mexico, Poland, and the US), over two-thirds remained statistically distinct from 0 after all adjustments (Figure 3, Table S2). In general, controlling for income had very little impact—only in Costa Rica, when comparing partnered to non-partnered adults, did adjusting for income produce a statistically significant change—and the estimate of difference in rates of grandparents coresidence lowers from a 3.8 (SE 0.015) to 2.3 (SE 0.016) percentage point difference and becomes statistically insignificant. Controlling for socio-demographic variables along with income explained the gap in grandchild coresidence in Croatia for both partnership and ADL status and in Mexico for partner status. In Poland and the US, adjustments for these variables did not explain any gaps (or lack of gaps).

**Figure 4.**
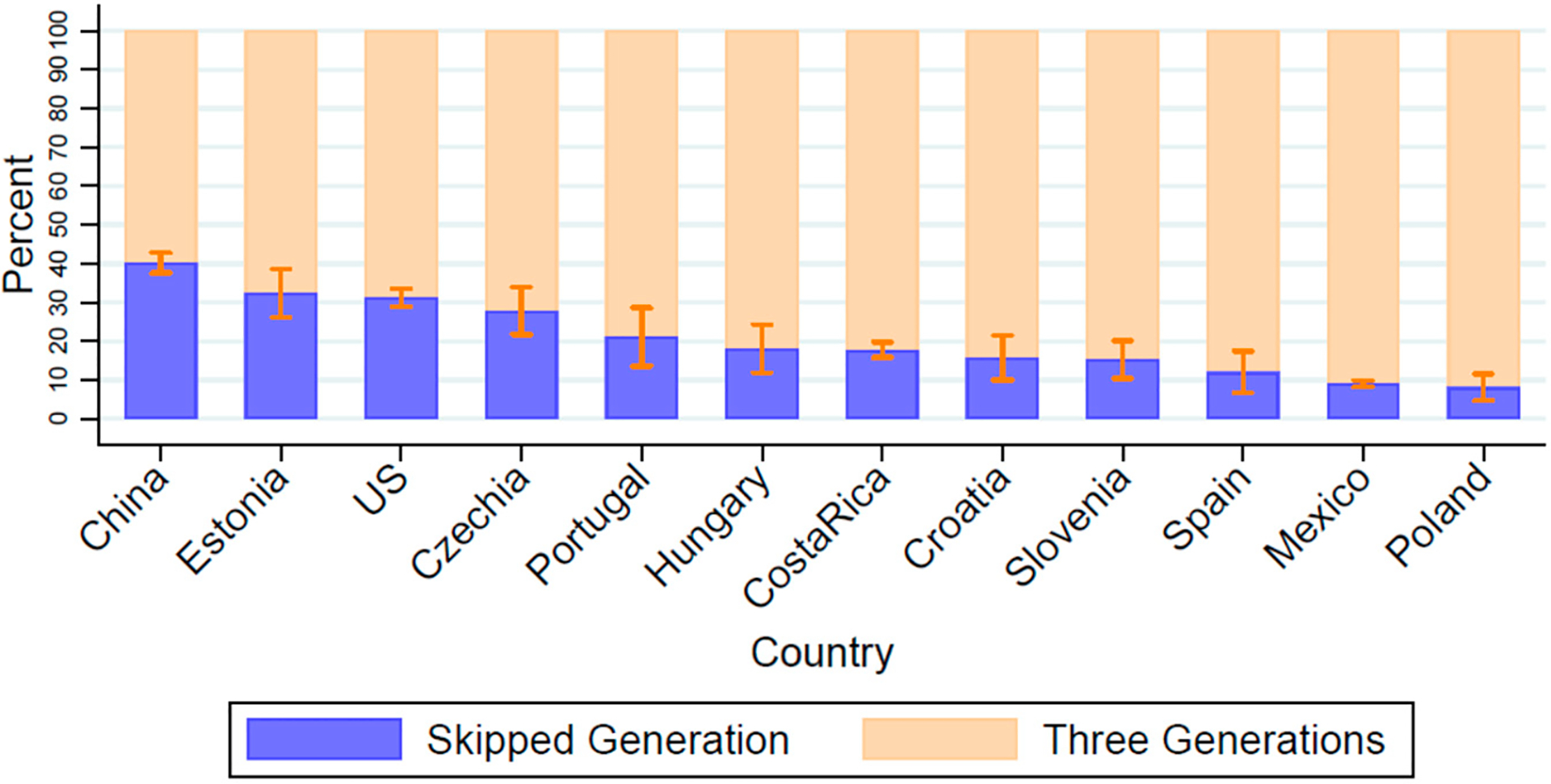
The majority of older adults who coreside with grandchildren are in three-generation households. Samples include older adults ages 55+ years who coreside with grandchildren. Countries with samples of over 100 older adults coresiding with grandchildren. Skipped Generation indicates none of the older adult’s children or children-in-law are present in the household. Data from the Gateway to Global Aging Studies; Grandchild coresidence calculated by the authors. Population weights applied. Data from 2015 with the following exceptions: China 2018, Costa Rica 2005 & 2011, Hungary 2011, Mexico 2012, Netherlands 2013, US 2014. See Supplementary Table S2 for sample sizes, exact rates and confidence intervals.

We limited our estimates of household composition to the 12 countries that had a sample of over 100 individuals with a coresident grandchild. In all countries, three-generation households were more common than skipped-generation households, but there was a wide degree of variation in the percentage of middle-aged and older adults with coresident grandchildren living in skipped-generation households. In China, 40.2% (CI 37.6–42.8%) of middle-aged and older adults with coresident grandchildren were living in skipped-generation households where the middle generation (child or child-in-law/grandchild’s parent) was not present in the household (Figure 4, Table S3). Estonia and the US had almost a third of middle-aged and older adults with coresident grandchildren in skipped generation households (32.4% CI 26.1–38.6% and 31.3% CI 26.1–38.6%, respectively), and Mexico and Poland had less than 10% (9% CI 8.2–9.9% and 8.1% CI 4.7–11.6%, respectively).

**Figure 5.**
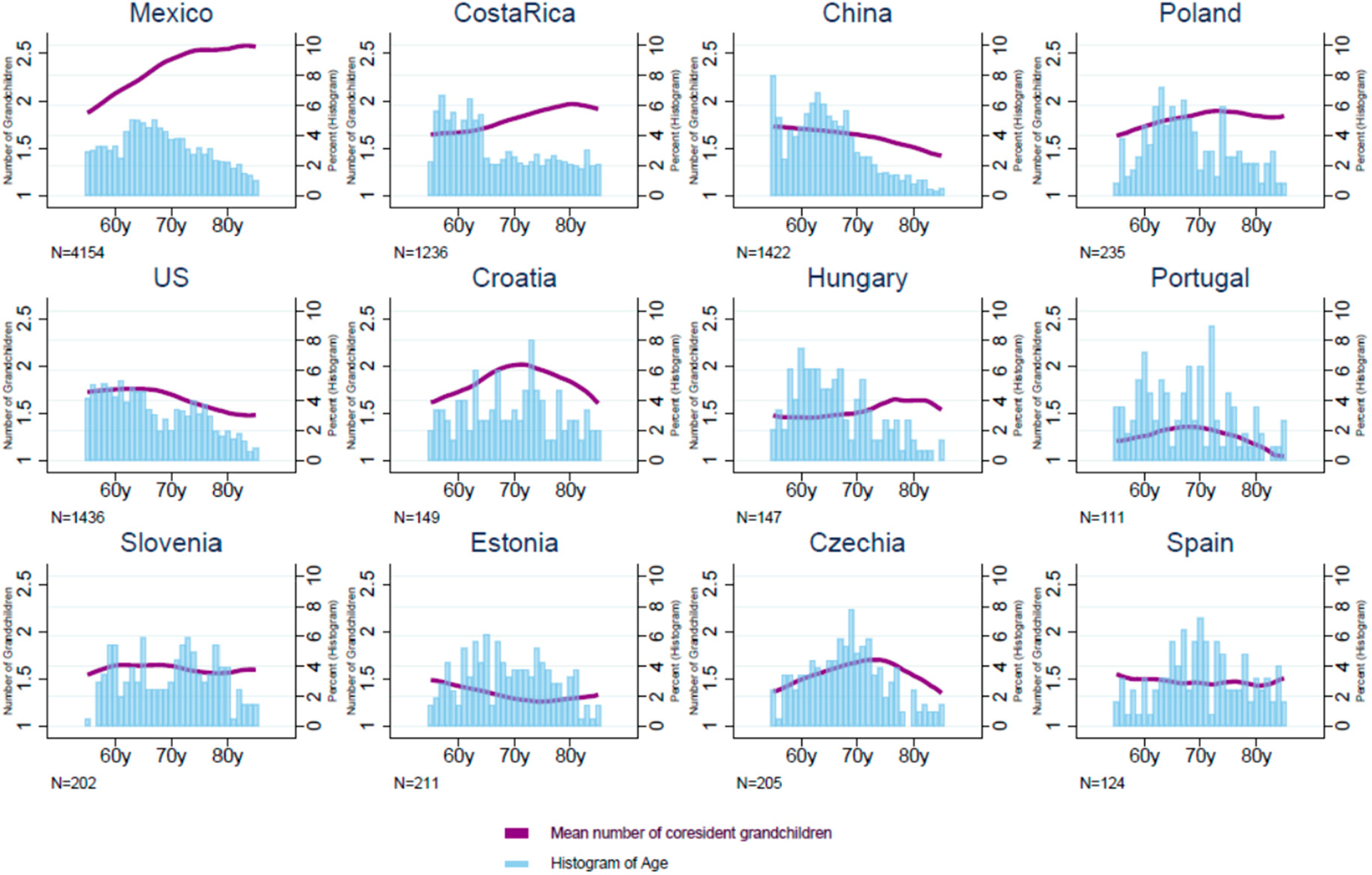
Mean number of coresiding grandchildren among adults with coresiding grandchildren. Adults ages 55–85 years who coreside with grandchildren. Data from the Gateway to Global Aging Studies; Grandchild coresidence calculated by the authors. Data from 2015 with the following exceptions: China 2018, Costa Rica 2005 & 2011, Hungary 2011, Mexico 2012, Netherlands 2013, US 2014. Countries with samples of over 100 individuals coresiding with grandchildren. Population weights applied.

In most of the same 12 countries, among middle-aged and older adults who had any coresiding grandchild, the mean number of coresident grandchildren was generally between 1.5 and 2 (Figure 5, Table S3). Lower average numbers of coresiding grandchildren were found in Estonia and Portugal (1.3 CI 1.3–1.4 and 1.3 CI 1.2–1.4, respectively). In Mexico, the average number of coresident grandchildren was higher and further increased from about 1.9 at 55 years to 2.7 at 85 years. In contrast, in China, Estonia, and the US, the average number of grandchildren declined by around 0.2 or 0.3 with age, while these trends were rather flat in Slovenia and Spain (both hovering tightly near 1.5).

**Figure 6.**
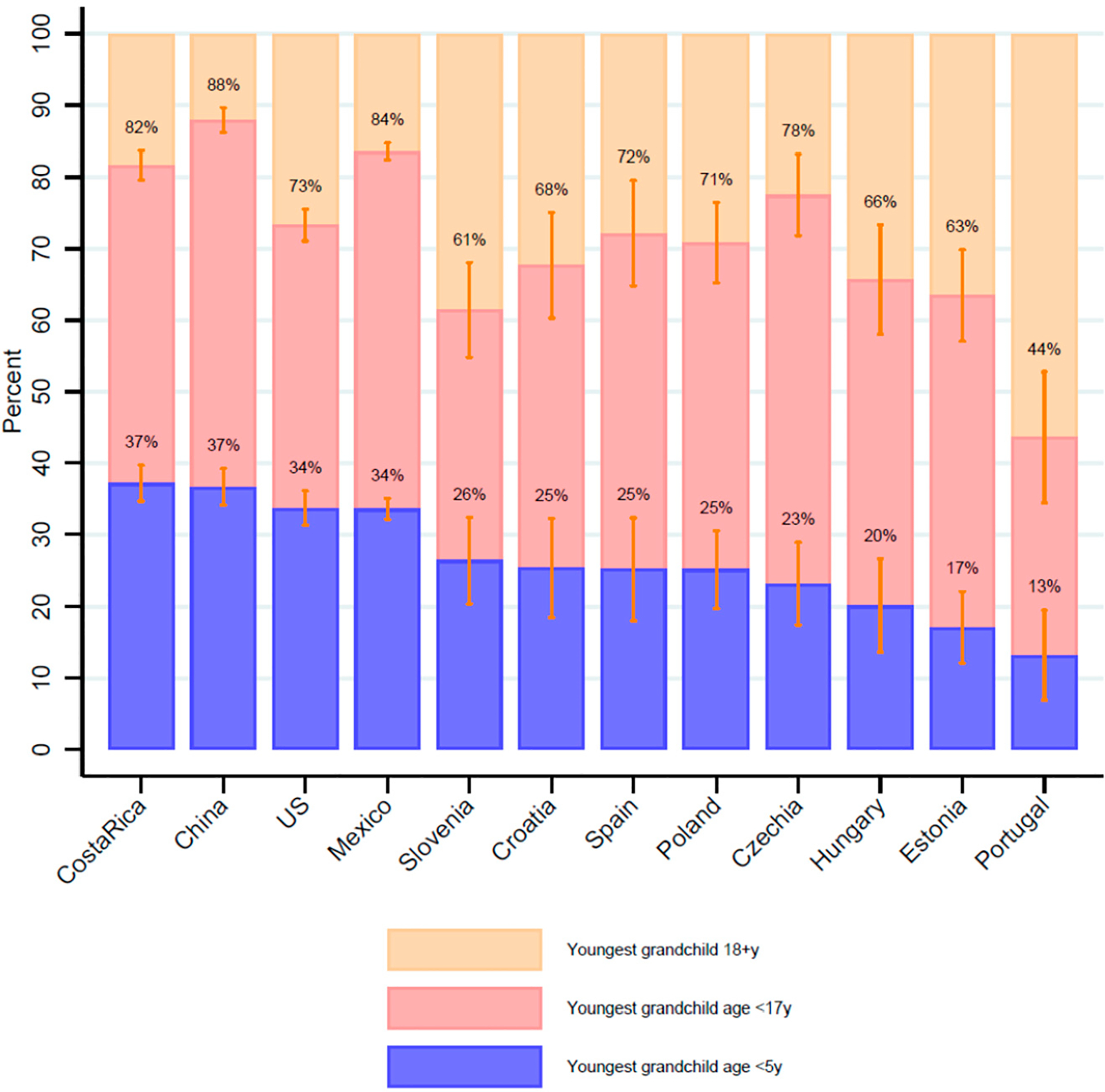
Among older adults who coreside with grandchildren, the age of the youngest grandchild is on a broad spectrum. Samples include older adults ages 55+ years who coreside with grandchildren. Countries with samples of over 100 individuals coresiding with grandchildren. Data from the Gateway to Global Aging Studies; Grandchild coresidence calculated by the authors. Population weights applied. Data from 2015 with the following exceptions: China 2018, Costa Rica 2005 & 2011, Hungary 2011, Mexico 2012, Netherlands 2013, US 2014. See Supplementary Table S2 for sample sizes, exact rates and confidence intervals.

We also found variation across the ages of the grandchildren in the 12 countries. In over a third of cases of middle-aged and older adults coresiding with grandchildren, there were no youth grandchildren in the households—only adult grandchildren ages 18 or older (Slovenia, Hungary, Estonia, and Portugal: Figure 6, Table S3). China had the smallest percentage (12.1% CI 10.4–13.8%) of coresidence with only adult grandchildren among middle-aged and older adults who had any coresiding grandchildren. Over a third of middle-aged and older adults with coresiding grandchildren in Costa Rica, China, the US, and Mexico had a grandchild age 5 years or less in the household. This was close to a quarter in five additional countries (Slovenia, Croatia, Spain, Poland, and Czechia). Only Estonia and Portugal had rates less than 20% of the youngest grandchild being age five or less among those who coresided with grandchildren.

**Figure 7.**
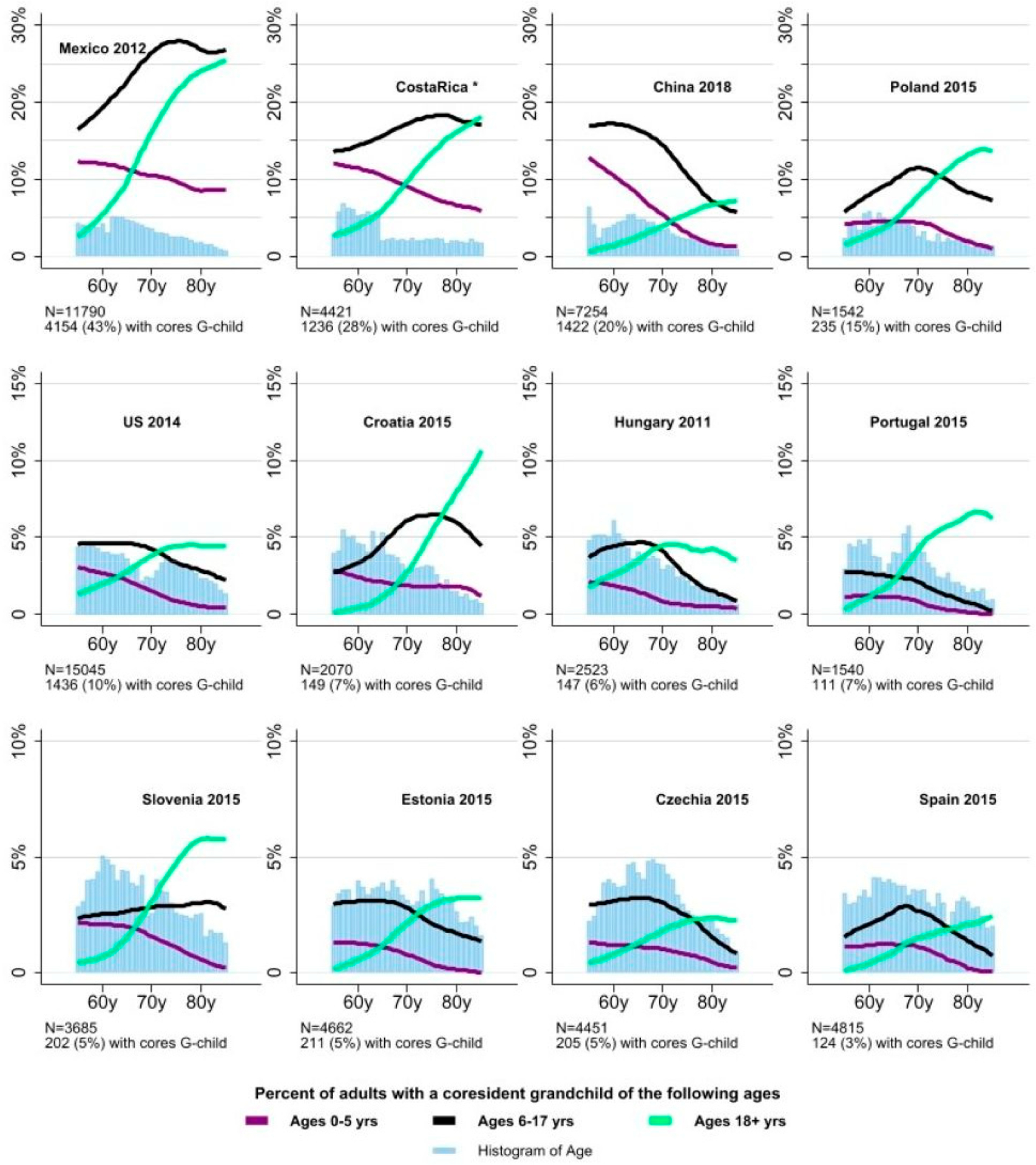
Percentage of adults coresiding with grandchildren of different ages. Adults ages 55–85 years. Data from the Gateway to Global Aging Studies; Grandchild coresidence calculated by the authors. * 2011 for those younger than age 65; 2005 for those 65 and older. Countries with samples of over 100 individuals coresiding with grandchildren. Population weights applied.

We further examined coresident grandchild age in light of the age of the middle-aged and older adults. We limited the ages of the middle-aged and older adult to be 55–85 years since there are fewer middle-aged and older adults at the tail of the distribution. For this reason, the percentage of grandparent–grandchild coresidence reported in this figure does not align with that reported in Figure 1, which included all adults 55+ years. With adult age as the running variable, we estimated the percentage with coresident grandchildren of certain age groups: 0–5, 6–17, and 18+ years (Figure 7). Not surprisingly, at older ages the percentage of older adults with the youngest grandchildren declined as the grandchildren were also aging.

The pattern over age was more varied for rate of grandchild coresidence ages 6–17 (Figure 7). In some countries it is primarily a decline (e.g., Portugal), while other countries had an increase and then decrease (e.g., Poland). The peak of the rate of coresident grandchildren ages 6–17 also varies, for instance, in Spain, it was prior to age 70; in Poland, it appeared to be at 70; and in Mexico, Costa Rica, and Croatia, it was after age 70.

The rate of adult grandchild coresidence typically increased with the age of the older adult, but this increase was more notable than the reduction in the rate of coresidence with grandchildren ages 0–5 (Figure 7). Adult grandchildren could become independent and live separately, but clearly that was not always the case. In Portugal and Slovenia, the rate of coresidence of adult grandchildren among the oldest adults was markedly higher than the rates of the two categories of non-adult grandchildren, particularly at the oldest ages.

China appears unique in the shapes of the age graphs of grandchild coresidence (Figure 7). Though the dramatic drop over the age of the older adult in percentage of non-adult grandchild coresidence is similar to that of some countries (e.g., Hungary for 6–17-year-olds and Slovenia for 0–5-year-olds), in China this decline was not matched by a similar or more dramatic increase in the rate of adult grandchild coresidence. For all other countries, the rate of grandchild coresidence for adults at the oldest ages was above or at least approximates the maximum rate of coresidence with 6–17-year-olds. In China, the rate of adult grandchild coresidence is 10% lower than the maximum rate of coresidence with 6–17-year-olds.

## 5. Discussion

Using population-based data from 24 countries, we show that a notable percentage of middle-aged and older adults live with their grandchildren in many global settings, although prevalence estimates ranged from as high as 41% (Mexico) to as low as 0.1% (Sweden). The prevalence varied by respondents’ (grandparents’) socio-demographics as well as their reported difficulty with activities of daily living (ADLs). Across settings, respondents in the demographic category typically associated with more advantage (partnered, male, no ADL impairment, urban, above median education, and top income quartile) generally had a lower rate of grandchild coresidence than the corresponding disadvantaged group. However, the absolute within-country differences in grandchild coresidence rates across the advantaged and non-advantaged groups were not particularly dramatic and income did not play a major role in explaining the differences by advantage. Setting aside the comparison of top to bottom income quartiles, which only contain half the population (and thus we expect the gaps to be larger by examining extremes), the largest gaps are in Mexico: just above a third of those above median education coreside with grandchildren while among the less educated just below a half coreside with grandchildren. Many countries do not even have statistically significant differences in rates of grandchild coresidence by ADL status, an area where one might expect more heterogeneity due to the needs of the older adult. This finding indicates that a broad approach to three-generational family policy not tailored to any specific subpopulation may be a reasonable starting point in many locations.

There was, however, evidence of cross-national variation. Middle-aged and older adults in China had particularly distinctive patterns of grandchild coresidence vs. other settings. In China, 40% of households where a grandchild coresided with a grandparent are skipped-generation households (vs. 32%-8% in other settings). Migration for work is common, and many parents leave their children to live with be cared for by their parents (middle-aged and older adults) [21]. The other notable distinction found in the Chinese case is the steep decline in grandchild coresidence with age compared to the other countries in the study. This may be due to the one-child policy. Most middle-aged and older adults in China under this policy would only have had one grandchild. Even if all grandchildren in every global location had the same propensity to remain coresiding with grandparents upon reaching adulthood, that Chinese grandparents had fewer grandchildren means the resulting rate of any grandchild coresidence is lower. Additionally, the Chinese demographic shift to the one-child policy also resulted in a culture of nuclear families and living alone [22].

Also of note are Mexico and Costa Rica—both upper-income Latin American countries—with the highest and second highest rates (41% and 26%) in our study of grandchild coresidence among middle-aged and older adults. This may be expected since intergenerational coresidence reinforces women’s identity as mothers even when their children are grown and Latin American culture generally emphasizes maternalism [23]. Additionally, among a study of intergenerational coresidence in developing countries, Latin America was the only region that maintained intergenerational coresidence over time despite wealth gains [2]. Though there is a large amount of literature on migration in Mexico, our study does not find as high a percentage of skipped generation families among middle-aged and older adults coresiding with grandchildren as in China. This could be due the fact that emigrants from Mexico to the U.S. have historically been predominantly men, e, which would leave the female parent in the household with both child and grandparent. The presence of mothers in the household could result in a lower grandchild caregiving burden for Mexican grandparents than Chinese grandparents, but the number of grandchildren in the household is higher in Mexico than in China.

All of the European countries in this study are upper-income countries. Theory suggests that maintaining intergenerational households can be a response to restricted resources or negative events [16]. Thus, the finding that most European countries—with a few exceptions such as Poland—had low rates of grandchild coresidence among middle-aged and older adults is somewhat expected. Yet, among the countries with a non-negligible sample of respondents with coresident grandchildren, those in European countries had higher proportions of adult (vs. young) grandchild coresidence. This suggests that the role of the coresident grandparent in Europe pertains less to providing caregiving for young grandchildren than in non-European countries. The US, however, is the only upper-income country within the four countries that have the highest rates of coresidence with very young grandchildren (<5 yrs), suggesting caretaking is an important driver of grandchild coresidence in the US. Additionally, this higher rate may somewhat be due to a skipped generational household structure, for example, in response to parental drug use [1].

Our study has limitations. Conclusions from changes over age should be interpreted with caution due to the cross-sectional nature of the data. It is possible that the rates and patterns of grandchild coresidence for the oldest adults in this study will be distinct from those of the youngest cohorts when they are ages 80+. We note, however, that our findings for Mexico are in alignment with those found by Reynolds and Torres [24], a longitudinal study of older adults’ coresidence with grandchildren in Mexico. They illustrate that cross-sectional estimates of coresidence with grandchildren underestimate the percentage of middle-aged and older adults who have ever lived with grandchildren. Additionally, the countries we include are a small representation of the globe, but China, the US, and Mexico are among the top ten most populous countries in the world [25] indicating that our results capture a meaningful fraction of grandparents coresiding with grandchildren worldwide.

Future work can explore differences in rates of grandchild coresidence in other arenas beyond the six bivariate measures we chose. Continuous exploration of income and education, for example, could be insightful. Due to our study being very broad and covering many countries, our analyses were not detailed around family particulars. For example, we pool grandchildren and great-grandchildren, we do not detail the specific make-up of the household such as whether there are grandchildren from multiple children, and we do not consider the family structure of the middle generation (if partnered, for example). Future research should extend to additional regions such as South-East Asia and Africa. Nevertheless, this is the first study to provide population-level cross-sectional descriptions of grandchild coresidence among middle-aged and older adults from many countries across the globe.

Policy interventions to support older adults need to be aware of the dynamics beyond spousal or parent–child relationships. Future research for policy interventions will need to focus on how to support both the oldest and the youngest generations in the household. In most households with grandchildren, the grandchildren are not adults, meaning that they will need resources for care, perhaps precisely when older adults are also needing resources for care. Yet our findings also showed that adult grandchildren were not uncommon in households, indicating that they may also have the potential to contribute financially and provide more care than they need. More research is needed to examine if these living arrangements are primarily due to financial constraints or cultural norms.

Our measures of cohabitation do not reflect the full sphere of close relationships between grandparents and grandchildren. A line of research finds important care and resource sharing between generations who reside at short distances from one another [26–29], suggesting that the rates found in our study represent just a portion of families that may be influenced by policies targeting intergenerational relationships. In many societies—including those in Europe where rates of grandparent–grandchild coresidence were lowest—family ties extend beyond household walls [30,31]. Additional research is needed to determine if coresident relationships are particularly unique in contrast to very close but non-coresident family arrangements.

Our key findings—that grandchildren of many ages coreside with middle-aged and older adults of many ages—point to some gaps in the literature. Research has focused on the following two common situations of grandchild coresidence among middle-aged and older adults: (1) when parents migrate and leave children in care of grandparents (as in China and Taiwan) [32–35]) and (2) when parents are dependent on substances and become unable to care for their own children (as in the US [32,36–40]). Both of these scenarios maintain the grandparent in the caregiving role instead of examining how grandchildren may impact their grandparents in later years.

Research on the grandparent–grandchild dyad focuses on the impact of caregiving for young grandchildren on grandparents, regardless of coresidence. This research suggests that caring for a grandchild can improve health [41,42] and be emotionally rewarding [43]. On the other hand, grandchild care can also be exhausting [32,44] and has been shown to be associated with more depressive symptoms [45]. These studies, however, are naturally restricted to those individuals with sufficient cognitive and physical abilities to provide caregiving. It is unknown how grandparent–grandchild coresidence may impact grandparents when a grandparent transitions from providing care to needing care. Future work will also need to examine how coresidence with young children can influence middle-aged and older adults who are not providing caregiving, as well as examining how older grandchildren who are not directly receiving care influence their coresident grandparents’ well-being.

We conclude that grandparent–grandchild coresidence is prevalent across many global settings, albeit with a great deal of across and between-country heterogeneity. Future research should focus on grandchild coresidence as a potential factor influencing the health and well-being of middle-aged and older adults.

## Supplementary Material

supplementary material

**Supplementary Materials:** The following supporting information can be downloaded at: https://www.mdpi.com/article/10.3390/populations1020012/s1, Table S1: summary statistics; Table S2: margins from Logit: predictors of grandchild coresidence among older adults; Table S3: summary statistics for older adults with coresident grandchildren.

## Data Availability

The original data presented in the study are openly available in the following repositories [46–52].
