# Supplementary material for: Grandparent–Grandchild Coresidence Among Middle-Aged and Older Adults Around the Globe
Source: Populations (Basel). Author manuscript; Available in PMC 2026 Jun 6. (PMC12379973; doi:10.3390/populations1020012)
Supplement: supplementary material [file NIHMS2089245-supplement-supplementary_material.zip › Supplementary Materials/Replication Files For Grandparent-Grandchild Coresidence G2GAging/Raw Data Folder Structure Info/Folder Structure for saving raw data.pdf]

File structure – Folders named “Data – Copy” and “Documentation” are not necessary for running the do files.

- Gateway2GlobalAging
  - CHARLS
    - Data
      - Life History
      - Wave 1
      - Wave 2
      - Wave 3
      - Wave 4
    - Data - Copy
    - Documentation
  - CRELES
    - Data
      - __MACOSX
      - CRELES_RC_w1_V2
      - CRELES_RC_w2_V1
      - CRELES_w1
      - CRELES_w2
      - CRELES_w3
      - H_CRELES
    - Data - Copy
    - Documentation
  - HRS
    - Data
      - Crosswave Tracker File
      - Rand
    - Documentation
  - MHAS
    - Data
    - Data - Copy
    - Documentation
  - SHARE
    - Data
      - sharew1_rel7-1-0_ALL_datasets_stata
      - sharew2_rel7-1-0_ALL_datasets_stata
      - sharew3_rel7-1-0_ALL_datasets_stata
      - sharew4_rel7-1-0_ALL_datasets_stata
      - sharew5_rel7-1-0_ALL_datasets_stata
      - sharew6_rel7-1-0_ALL_datasets_stata
      - sharew7_rel7-1-1_ALL_datasets_stata
      - sharew8_rel1-0-0_ALL_datasets_stata
      - sharewX_rel7-1-0_gv_allwaves_cv_r_stata
      - sharewX_rel7-1-0_gv_linkage_stata
      - sharewX_rel7-1-0_gv_longitudinal_weights_stata
    - Data - Copy
    - Documentation
    - SHARE_computing_calibrated_weights_stata
      - Analysis
        - ado
        - SHARE Examples
          - data
