# Supplementary material for: Grandparent–Grandchild Coresidence Among Middle-Aged and Older Adults Around the Globe
Source: Populations (Basel). Author manuscript; Available in PMC 2026 Jun 6. (PMC12379973; doi:10.3390/populations1020012)
Supplement: supplementary material [file NIHMS2089245-supplement-supplementary_material.zip › Supplementary Materials/Replication Files For Grandparent-Grandchild Coresidence G2GAging/Read Me.docx]

Read Me

Set up your project folder with the following folders:

Replication Files For Grandparent-Grandchild Coresidence G2GAging

Stata Output

Graphs

Working Data

CHARLS

CRELES

HRS

MHAS
 SHARE

Download files from [Replication Files For Grandparent-Grandchild Coresidence G2Gaging](https://1drv.ms/f/s!Alx8DbDo9bmihNBGp5jmnkS6bzvuPw?e=EercpZ) or from the Journal website into that folder.

Prepare the Raw Data as described on the Gateway to Global Aging site using the file structure provided in “Replication Files For Grandparent-Grandchild Coresidence G2Gaging/Raw Data Folder Structure Info”

Update directories in the profile

Update Master.do with your paths and run
